# Psychometric validation of the Danish cancer caregiver roles and responsibilities scale

**DOI:** 10.1007/s11136-026-04234-8

**Published:** 2026-04-01

**Authors:** Iben Husted Nielsen, Christian Landbo, Tine Rosenberg, Karin Piil, Anders Tolver, Valerie Shilling, Mary Jarden

**Affiliations:** 1 Department of Hematology, Copenhagen University Hospital, Rigshospitalet, Copenhagen, Denmark; 2 Department of Haematology, Odense University Hospital, Odense, Denmark; 3 Department of Oncology, Copenhagen University Hospital, Copenhagen, Denmark; 4 School of Nursing, Faculty of Health Sciences, Curtin University, Western Australia Perth, Australia; 5 Statistics and Data Analysis, Danish Cancer Institute, DK- 2100 Copenhagen, Denmark; 6 Department of Clinical and Experimental Medicine, Brighton and Sussex Medical School, University of Brighton and University of Sussex, Brighton, UK; 7 Department of Clinical Medicine, University of Copenhagen, Copenhagen, Denmark

**Keywords:** Caregiving, Cancer, Psychometric performance, Validation, Quality of life

## Abstract

**Purpose:**

The purpose of this study was to translate and culturally adapt the Caregiver Roles and Responsibilities Scale (CRRS) into Danish and to conduct a psychometric analysis of its validity and reliability among informal caregivers of cancer patients in Denmark.

**Methods:**

The translation and cultural adaptation followed the Functional Assessment of Chronic Illness Therapy (FACIT) translation methodology. Cognitive debriefing interviews were carried out with ten native Danish-speaking caregivers to assess comprehensibility and relevance. Psychometric properties were evaluated using a survey, test-retest design, with data collected from 180 caregivers at baseline (t0) and a subsample of 80 caregivers at the second time point (t1), 10 days after baseline, to evaluate test–retest reliability using Intraclass Correlation Coefficient (ICC).

**Results:**

The Danish CRRS demonstrated good psychometric properties, with Cronbach’s α values exceeding the acceptable threshold of 0.70 for most subscales, indicating high internal consistency. ICC analysis showed strong test—retest reliability across all subscales (ICC ≥ 0.80) and excellent reliability for the total CRRS score (ICC = 0.91), confirming the measure’s reliability over time.

**Conclusion:**

The Danish CRRS demonstrated good validity and test—retest reliability. The CRRS is now available for Danish-speaking individuals to use in both clinical cancer care and research for assessing the impact of cancer on the lives and wellbeing of informal caregivers.

## Introduction

Informal caregivers are essential in providing practical and emotional support to people living with cancer, significantly influencing patients’ quality of life (QoL) and treatment outcomes [1, 2]. However, the responsibilities associated with caring for a person with cancer can be demanding, often leading to a decline in caregivers’ QoL and risk of psychological stress, depression, and anxiety [3–7]. As advancements in cancer treatment prolong survival for many patients and increasingly shift treatment toward home-based delivery, caregivers may face growing demands, further challenging their resources and ability to provide adequate support [8]. Existing evidence show that informal caregivers undertake a wide range of tasks and responsibilities, which evolve in response to the patient’s changing needs, disease progression, and course of treatment [9–11]. Moreover, caregivers of cancer patients often juggle multiple roles, such as childcare, employment, and caring for other family members, further complicating their capacity to manage these challenges [5, 12]. Many caregivers report dissatisfaction with recognition from healthcare professionals, indicating a need for improved communication and engagement with healthcare professionals [13]. Ensuring caregivers’ wellbeing is essential for them to support patients effectively and prevent chronic stress and physical strain [3, 14]. Although several self‑reported instruments exists for assessing caregivers, two systematic reviews indicate that many have not undergone rigorous psychometric validation and often focus primarily on the psychological aspects of caregiver burden [11, 15]. In Denmark, only a few validated Danish‑language caregiver instruments are available, and none fully capture the multifaceted impact of caregiving across the cancer trajectory [16, 17].

To address this gap, the Caregiver Roles and Responsibilities Scale (CRRS) was developed to capture broader dimensions of the caregiving experience, including changes in roles and responsibilities as well as practical and financial consequences. The CRRS comprises 41 core items and five subscales covering dimensions of *Support and Impact*,* Emotional Wellbeing*,* Lifestyle*,* Self-care*, *Financial Wellbeing*, and one standalone scale *Jobs and Career* which has demonstrated good psychometric properties in UK-based cancer caregivers [18]. Despite the increasing integration of patient-reported outcome measures in Danish clinical practice [19], caregivers’ wellbeing is not routinely assessed. The CRRS therefore represents a promising tool for both research and clinical practice. A validated Danish version of the CRRS is warranted to address these gaps and to support a more comprehensive assessment of caregiving.

The objective of this study was to translate and culturally adapt the CRRS into Danish and to evaluate its psychometric properties, specifically assessing internal consistency, test – retest reliability, and exploratory associations with relevant caregiver characteristics.

## Methods

### Study design

This cross-cultural validation study was conducted at the Departments of Oncology and Hematology at Copenhagen University Hospital and Odense University Hospital, Denmark. The study involved two steps:

1. Translation, adaptation, and pilot testing
2. Analysis of psychometric properties. The translation and cross-cultural adaption adhered to the Functional Assessment of Chronic Illness Therapy (FACIT) translation methodology [20]. Psychometric properties were assessed using a non-interventional test-retest design with survey data collected at two time points, baseline (t0) and follow-up (t1). Permission to use the original questionnaire was granted by its developer, Shilling et al. [18].

### Translation and cultural adaptation

#### Translation and adaptation

**Fig. 1:**
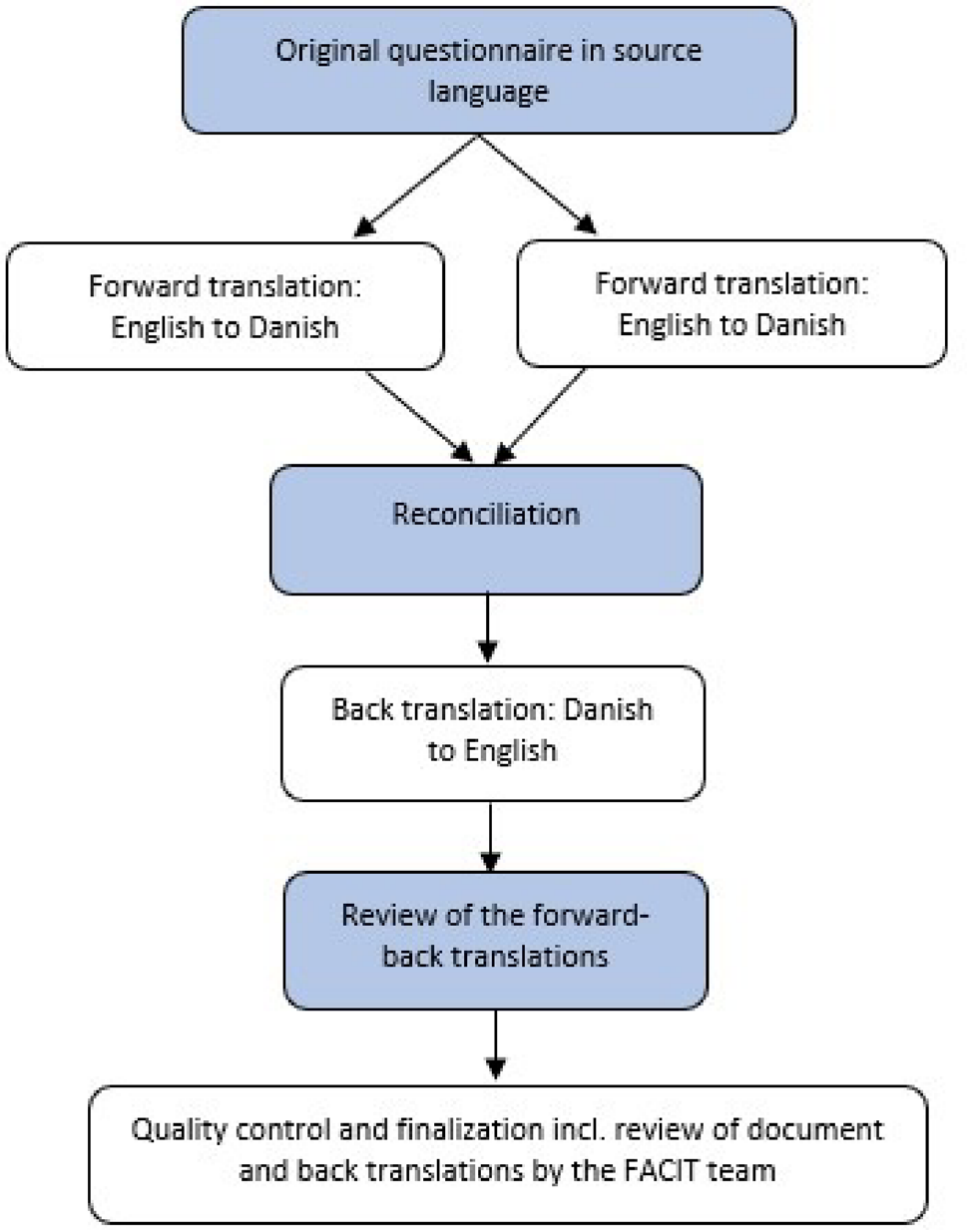
Translation pathway for the CRRS questionnaire

The translation and cultural adaptation from English to Danish involved both forward and back translations, review, and pilot testing (Fig. 1) and was completed through the following steps:

First, two bilingual healthcare professionals translated the CRRS from English to Danish, prioritizing meaning over literal translation. Next, a third bilingual person, blinded to the original English version, reconciled the two translations into one, adjusted as needed to enhance clarity and suitability. To further ensure quality, a native English professional translator with strong Danish skills, uninvolved in prior steps and without access to earlier translations, performed a verbatim back translation of the reconciled version into the original source language.

The entire research team reviewed and discussed the results, highlighted difficult-to-translate items in a report to FACIT team to ensure equivalence with the original source. Final adjustments regarding wording, intent, and cultural relevance were made. In cases where direct translation was not possible, minor wording adjustments were made to align with Danish language nuances. Finally, the Danish version was formatted into appropriate templates and approved by the FACIT organization for pilot testing.

#### Pilot testing

To assess the comprehensibility and readability of the Danish CRRS, a pilot test was conducted with 10 participants. Participants were ≥ 18 years, native speakers of the Danish language, and informal caregivers of adult persons with cancer undergoing cancer treatment or previously treated for cancer. Participants were purposively sampled to include individuals with relevant caregiving experience and diversity in age, gender, and relationship type. Participants first completed the Danish CRRS, after which a cognitive interview lasting 50–60 min was conducted by telephone or online by one of two researchers (IHN or HE). A structured interview guide provided by the FACIT translation team was used to prompt participants to reflect on each item’s clarity, relevance, and comprehensibility. Participants were encouraged to use a think-aloud approach and explain each item in their own words in response to prompts such as “What did you think about when answering this item?” and “What does the phrase mean in this item?”. Feedback was documented and transcribed item by item, translated from Danish to English, and systematically reviewed by the research team (IHN, HE, MJ) to determine whether items should remain unchanged or be modified. Necessary revisions were incorporated and subsequently submitted to the FACIT translation team for approval. The final version was approved by the original developer and by the FACIT organization in November 2020.

### Psychometric validation and reliability

#### Sample and data collection

Participants were consecutively recruited from inpatient and outpatient oncology and hematology departments using convenience sampling. Inclusion criteria were: (1) an informal caregiver aged ≥ 18 years of a person aged ≥ 18 years with any hematologic or oncologic cancer, identified by the patient as being a close caregiver; and (2) sufficient Danish language skills.

There are no established uniform guidelines for sample size when evaluating internal consistency or test-retest reliability in patient‑reported outcome measures [21]. However, psychometric literature suggests that larger samples improve the stability of internal consistency estimates and that test-retest analyses typically require around 50 participants with an interval of 10–14 days between assessments [22, 23]; in this study, a 10 day interval was used. Based on these considerations and to account for non-responders, 290 questionnaires were distributed, 190 of which included two copies of the questionnaire with instructions to complete one at baseline and the other at follow-up.

Data collection took place from June 2021 to November 2021. Eligible participants were approached while assisting patients during hospital visits. Staff members explained the purpose of the study and provided study materials to those who gave informed verbal consent. Study materials included an information letter describing the study aim and methods, a paper copy of the CRRS questionnaire including demographic questions, and a pre-paid return envelope. Completion and return of the survey were considered as consent to participate. Participants were encouraged to complete the questionnaire either during the hospital visit or at home. During the COVID-19 pandemic, when caregivers had limited access to the hospital, patients were provided with study information to share with their informal caregivers. All participants completed the CRRS along with sociodemographic information, which included age, gender, highest educational level, current employment status, relation to the patient, caregiving duration, and patient’s diagnosis and treatment. For those who completed the CRRS retest after 10 days (t1), an additional question assessed self-perceived changes in caregiving responsibilities since t0, with five response options: ‘much worse’, ‘worse’, ‘unchanged’, ‘better’, ‘much better’.

#### Measurement

The CRRS comprises 41 core items, including five subscales: *Support and Impact*, *Lifestyle*, *Emotional Health and Wellbeing*, *Self-care*, and *Financial Wellbeing*. Additionally, the CRRS includes two binary (yes/no) response items regarding whether caregivers have stopped working due to their caregiving responsibilities or intend to return to paid employment. Only participants currently employed respond to the standalone scale, *Jobs and Career* (Subscale 6).

Participants are instructed to respond to each item using a five-point Likert scale ranging from ‘Not at All’ to ‘Very Much,’ with a recall period of seven days. Sum scores are calculated by assigning numeric values (0 to 4) to the Likert responses (ranging from ‘Not at all’ to ‘Very much’), with reverse scoring applied to specific items as outlined in the CRRS v1 Scoring Guidelines [24]. The total score is computed based on subscale 1–5 scores, which excludes three standalone items (Sp9, CS57, CS1), resulting in a total score range of 0-152. However, these three standalone items (Sp9, CS57, CS1) can be added if desired for a CRRS-41 total score, which is the case for this study (possible range: 0-164). The *Jobs and Career* subscale score is not included in any total score. For both subscale and total scores, higher values indicate better QoL. In the original CRRS development study internal consistency, measured by Cronbach’s α, was excellent for the full scale (α = 0.92 at baseline and α = 0.94 at the second measurement). The subscales *Emotional Health and Wellbeing* (α = 0.87), *Lifestyle* (α = 0.87), *Support and Impact* (α = 0.82), *Self-care* (α = 0.75), and *Financial Wellbeing* (α = 0.78) all demonstrated acceptable to good internal consistency [18].

#### Statistical analysis

According to the scoring manual, missing data within subscales was handled by prorating scores when more than 50% of the items were answered. To ensure overall data completeness, a total score was computed only for participants who completed at least 80% of all items (33 of 41).

Psychometric properties were examined using relevant measures of internal consistency, validity, and test-retest reliability. Internal consistency was evaluated using Cronbach’s α on items grouped by their assigned subscale. Cronbach’s alpha values of ≥ 0.7, ≥ 0.8, and ≥ 0.9 were considered acceptable, good, and excellent reliability, respectively [25]. To evaluate test-retest reliability, the Intraclass Correlation Coefficient (ICC) was calculated using a one-way random effects model based on total scores and subscale sum scores at t0 and t1, with ICC ≥ 0.80 indicating good or excellent reliability across test administrations. To assess stability and accuracy of measurements over time, the Wilcoxon signed rank-test was performed to compare subscale scores and total CRRS scores between t0 and t1. It was anticipated that no significant differences would be observed between baseline and follow-up, supporting the measure’s stability over time. Spearman’s correlation coefficient (ρ) was calculated to explore the relationship between self-perceived caregiver responsibility change scores on a five-point Likert scale, “Much worse (0)” to “Much better (4)” and observable changes in subscale sum scores and total CRRS scores at t1 compared to t0. Spearman’s ρ results was interpreted based on the strength and direction of the association, with values closer to ± 1 indicating stronger relationships and values near 0 indicating weaker or no association. These analyses were conducted as post hoc exploratory group comparisons to examine whether CRRS subscale scores varied across relevant categorical caregiver and patient characteristics. Associations between categorical variables and subscale scores were analyzed using non-parametric tests (Mann-Whitney and Kruskal-Wallis test for multiple group comparisons). P-values below 0.05 were considered statistically significant. For post-hoc pairwise comparisons, we used Dunn’s test with Bonferroni correction. We emphasize the exploratory nature of the analysis and the risk of some false positive findings (type I errors), due to the number of sub-scales and statistical tests presented. All analysis were carried out using R Statistical Software v. 4.2.2 [26].

### Ethical considerations

This study adhered to the principles of the Declaration of Helsinki [27] and was approved by the Regional Committees of Southern Denmark (no.:20/200725) and the Capital Region (no: p-2024-15600).

Informal caregivers of cancer patients are at risk of developing their own symptoms and concerns, and therefore deserve attention and support as a distinct group. Moreover, prior research indicates that caregivers appreciate and benefit from participating in research [28]. To minimize burden, caregivers were not approached on the day of the patient’s diagnosis or during periods of critical medical instability. All data, including sociodemographic and medical information, were self-reported, and researchers had no access to patients’ medical records.

## Results

### Translation and cultural adaption

The translation process led to a revision of the heading in subscale five from “Financial Wellbeing” to the Danish term “Financial Security” (Danish: Finansiel sikkerhed). However, back translation revealed that this term did not fully convey the original intent and was culturally inappropriate due to the Danish tax-funded healthcare and welfare systems providing social insurance, free medical treatment, and financial support. Therefore, it was revised to the more neutral term “Economic Situation” (Danish: Økonomisk situation). Translating “coping” (item CS24) was also challenging without a specific context. The research group added the phrase “with my situation”, resulting in a revised item: “People are showing interest in how I am coping with my situation” (Danish: Folk viser interesse for, hvordan jeg håndterer min situation). In conclusion, two changes were made in the Danish translation of the CRRS.

### Cognitive debriefing interviews for pilot-testing

A total of 10 cognitive interviews were conducted between May 2020 and August 2020, in line with the FACIT translation methodology [20]. Due to COVID-19 restrictions during that period, interviews were conducted by telephone or video call. Eight women and two men, aged 29 to 73, were included. Most were spouses (*n* = 7), with one sibling, one child, and one parent. The estimated time to complete the questionnaire was 15–20 min. Overall, all participants perceived the questions, instructions, and answer options as clear, with no offensive wording or issues identified. However, some provided comments on specific items. For instance, the item ‘I find comfort in my faith or spiritual beliefs’ (Sp9) was considered irrelevant by some due to their personal perspectives on the topic, leading them to select the answer ‘not at all’. Moreover, non-spousal caregivers noted that some questions, particularly those addressing financial impact, were less relevant, as they did not share finances with the patient. Although concerned about the patient’s financial wellbeing, they had no way to address this concern in the questionnaire.

Some participants noted that the word ‘responsibilities’ (Danish: forpligtelser) emphasizes external expectations, such as legal or moral obligations, rather than the commitment driven by love. Since both nuances are encompassed in the Danish term and no better alternative was found, it was retained. The cognitive interview transcripts were translated into English and submitted to the FACIT translation team, and the final version was approved by both the developer and FACIT team.

### Psychometric evaluation of the Danish version of CRRS

#### Participant characteristics

The baseline sample (t0) included 180 participants, of whom 80 completed the follow‑up assessment (t1) used for the test–retest analysis, resulting in a 44% follow-up response rate. Most participants were female (61%) and were spouses or partners (71%). Ages ranged from 27 to 85 years (mean 60 years). In 37% of cases, participants cared for patients diagnosed within the past year. The majority were caregivers of patients with hematologic cancer (51%), followed by those caring for patients with breast cancer (17%). Demographic and clinical characteristics are summarized in Table 1.

**Table 1:** Categorical variables and population distribution

Categorical variables	Population *n* = 180 (100%)*
*Age group, n (%)*	
< 50	35 (19.4%)
50–64	68 (37.8%)
≥ 65	63 (35.0%)
*Sex, n (%)*	
Male	69 (38.3%)
Female	109 (60.6%)
Relation, n (%)	
Partner	127 (70.6%)
Child	30 (16.7%)
Parent	10 (5.6%)
Friend	1 (0.6%)
Other	10 (5.6%)
Education, n (%)	
Elementary	19 (10.6%)
Further	26 (14.4%)
University	123 (68.3%)
Occupational degree	7 (3.9%)
Other	3 (1.7%)
*Employment status, n (%)*	
Employed / self-employed / sick leave	104 (57.8%)
Unemployed	3 (1.7%)
Retired	68 (37.8%)
*Stopped working due to caregiver role, n (%)*	
Yes	7 (3.9%)
No	93 (51.7%)
*Caring for a person with, n (%)*	
Head/Neck	5 (2.8%)
Gastrointestinal	9 (5.0%)
Hematological	91 (50.6%)
Gynocological	2 (1.1%)
Renal	8 (4.4%)
Breast	30 (16.7%)
CNS	6 (3.3%)
Respiratory	12 (6.7%)
Other	13 (7.2%)
*Caring for a person receiving treatment, n (%)*	
Yes. medicinal treatment	73 (40.6%)
Yes. radiotherapy	101 (56.1%)
No. ambulatory	3 (1.7%)
*Caring for a person hospitalized, n (%)*	
Yes	32 (17.8%)
No	141 (78.3%)
*Time from diagnosis, n (%)*	
< 1 year	65 (36.1%)
1–2 years	18 (10.0%)
> 2 years	94 (52.2%)
*Caregiver for more than one, n (%)*	
Yes	24 (13.3%)
No	151 (83.9%)

*Percentages are calculated based on the total baseline population; remaining percentages represent missing values

#### Internal consistency of the Danish CRRS

Cronbach’s α was calculated for each subscale as well as for the total CRRS score using results from the baseline (t0) assessments (Table 2).

**Table 2:** CRRS-41 total score and subscale Cronbach’s α and ICC score

Subscale	Alpha (95% - CI)	ICC (95% - CI)
Support and Impact	0.83 (0.78–0.86)	0.83 (0.74–0.88)
Lifestyle	0.88 (0.86–0.91)	0.83 (0.75–0.89)
Emotional health and wellbeing	0.87 (0.85–0.90)	0.88 (0.82–0.92)
Self-care	0.68 (0.61–0.75)	0.80 (0.71–0.87)
Financial wellbeing	0.76 (0.67–0.79)	0.79 (0.70–0.86)
Job and career	0.54 (0.34–0.66)	0.78 (0.63–0.88)
CRRS total score	0.91 (0.89–0.92)	0.91 (0.86–0.94)

The total CRRS score demonstrated excellent internal consistency (Cronbach’s α = 0.91, 95% CI 0.89–0.92). Internal consistency was good for the subscales *Support and Impact* (α = 0.83, 95% CI 0.78–0.86), *Lifestyle* (α = 0.88, 95% CI 0.86–0.91), and *Emotional Health and Wellbeing* (α = 0.87, 95% CI 0.85–0.90). The *Financial Wellbeing* subscale showed acceptable internal consistency (α = 0.76, 95% CI 0.67–0.79). The subscales *Self-care* and *Job and Career* did not meet the minimum threshold of 0.70 for acceptable internal consistency, with Cronbach’s α = 0.68 (95% CI 0.61–0.75) and α = 0.54 (95% CI 0.34–0.66), respectively.

#### Test-retest reliability

ICC was calculated for the total CRRS score, and the subscale sum score results from participants that had completed the follow-up questionnaire (*n* = 80). Total CRRS score illustrated excellent reliability (ICC = 0.91, CI 95% 0.86–0.94) between t0 and t1.

Four of the subscales fulfilled the threshold ICC ≥ 0.80, while Subscale 5, *Financial Wellbeing* (ICC = 0.79, CI 95% 0.70–0.86) and Subscale 6, *Job and career* (ICC = 0.78, CI 95% 0.63–0.88) fell just below the threshold. (Table 2). No statistically significant difference between mean scores at t0 and t1 were found for the total CRRS score or any subscale scores, except for *Job and Career* (mean difference − 1.08, *p* = 0.004) (Table 3).

**Table 3:** Subscale and CRRS-41 scores at T0 and T1, including score changes, associations with self-perceived change, and floor/ceiling effects

Scale	Baseline population, t0		Two-time responders, t0		Two-time responders, t1		Change between t0 and t1**	Self-perceived change and change in score from t0 and t1 ***	Floor and ceiling effect****
	*n*	mean (SD)	*n*	mean (SD)	*n*	mean(SD)	*p*-value	ρ (*p*-value)	floor, *n* (%) / ceiling, *n* (%)
Support and impact *(range 0–24)*	180	14.5 (5.2)	80	14.2 (5.5)	80	13.6 (5.5)	0.10	0.02(0.85)	106 (9.9) / 270 (25.2)
Lifestyle *(range 0–44)*	180	27.6 (8.8)	80	29.5 (8.8)	80	29.6 (8.8)	0.62	0.30 (0.01*)	160 (8.1) / 562 (28.6)
Emotional health and wellbeing *(range 0–36)*	180	24.1 (8.0)	80	25.3 (7.5)	80	25.6 (8.3)	0.33	0.18 (0.12)	148 (9.2) / 541 (33.6)
Self-care *(range 0–24)*	180	18.8 (3.3)	80	19.2 (3.2)	80	18.9 (3.3)	0.24	0.21 (0.06)	19 (1.8) / 514 (47.7)
Financial wellbeing *(range 0–24)*	180	19.7 (4.2)	80	20.3 (4.0)	80	19.9 (3.9)	0.24	0.08 (0.50)	61 (5.7) / 702 (65.9)
Job and Career *(range 0–28)*	102	22.8 (3.3)	41	22.8 (3.8)	41	21.7 (4.2)	0.004*	0.06 (0.73)	49 (6.9) / 437 (61.6)
Total CRRS-41 Score *(range 0-164)*	180	109.8 (21.5)	80	113.9 (21.2)	80	113.1 (23.6)	0.23	0.31 (0.01*)	

* Statistical significance (*p* ≤ 0.05)

**Change between t0 and t1 scores were tested with Wilcoxon signed-rank test to assess statistical significance.

*** Relationship between self-perceived change in caregiver responsibility (5-level Likert scale, 0 = much worse, 4 = much better) and actual change in CRRS subscale and total scores between t0 and t1 was assessed with Spearmans correlation test.79 participants replied to the self-perceived change item.

**** Negatively stated items is reversed according to *CRRS v1. Administration and Scoring Manual*. The items are cs2, co7, co9, co2, co5, ch4, ch5, co8, co6, ch11, co10, ge1, ch6, ch8, ch7, cr3, cr6, ft3, cf2, cf3, cf4, ce3, ce4, ce7, ft9.

All reversed items are to be interpreted as 0 = Very much and 4 = Not at all

Among the 80 participants who completed the follow-up assessment, 79 responded to the self-perceived change scores. The majority reported no change in self-perceived caregiver responsibilities (*n* = 48, 61%), while 18% (*n* = 14) experienced some worsening and 14% (*n* = 11) noted some improvement. Only 1% (*n* = 1) reported a significant worsening of caregiver responsibilities, and 6% (*n* = 5) reported significant improvement. A statistically significant positive relationship between self-perceived change and a change in total CRRS score from t0 to t1, was observed (ρ = 0.31, *p* = 0.01). Furthermore, Subscale 2, *Lifestyle* also illustrated a statistically significant positive relationship between self-perceived change and difference in subscale sum score from t0 to t1 (ρ = 0.30, *p* = 0.01) (Table 3).

#### Associations between subscale scores and categorical variables

The CRRS outcomes are presented with mean (SD) for each categorical variable in Table 1 Five key characteristics showed statistically significant associations with four of the subscale scores (Table 4).

**Table 4:**
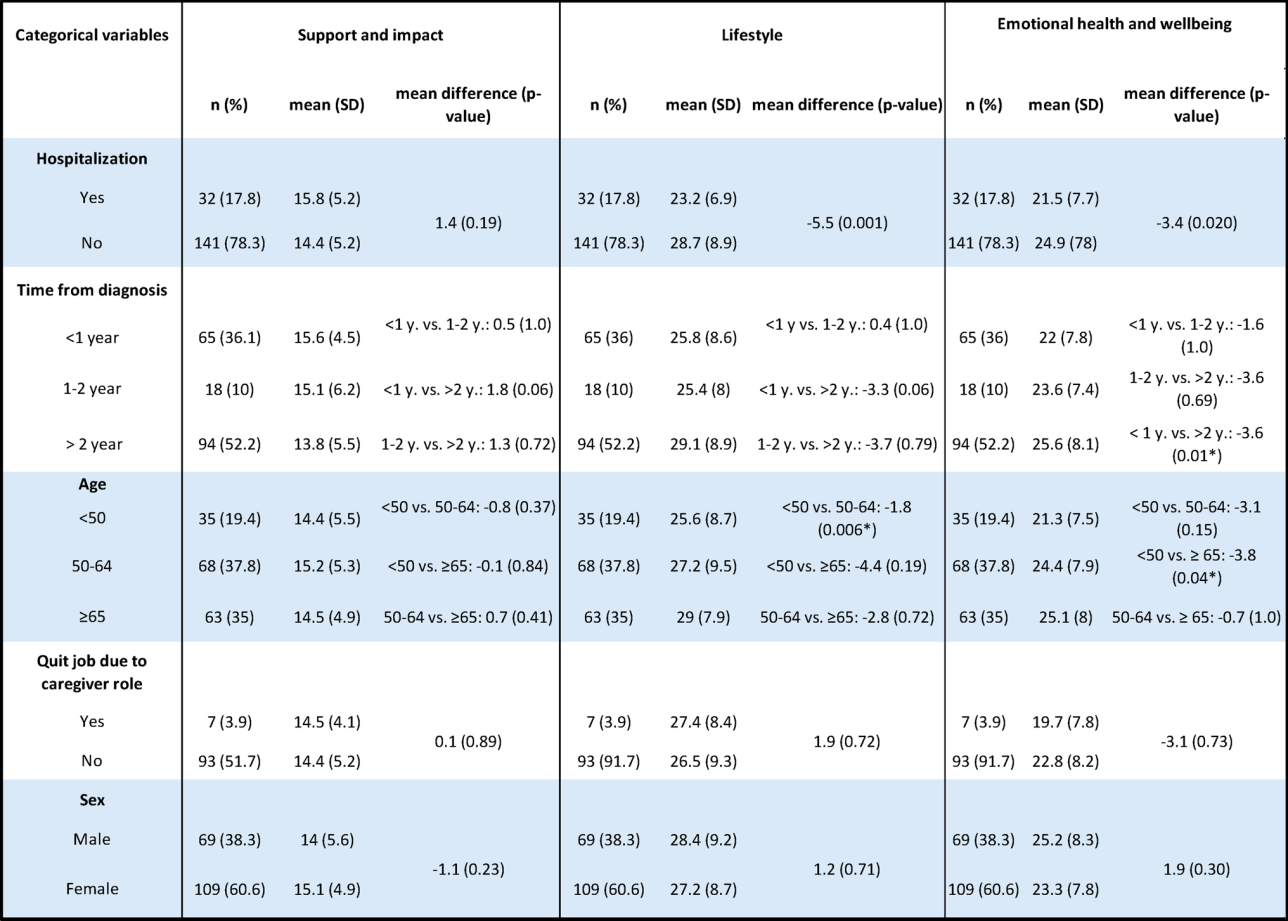

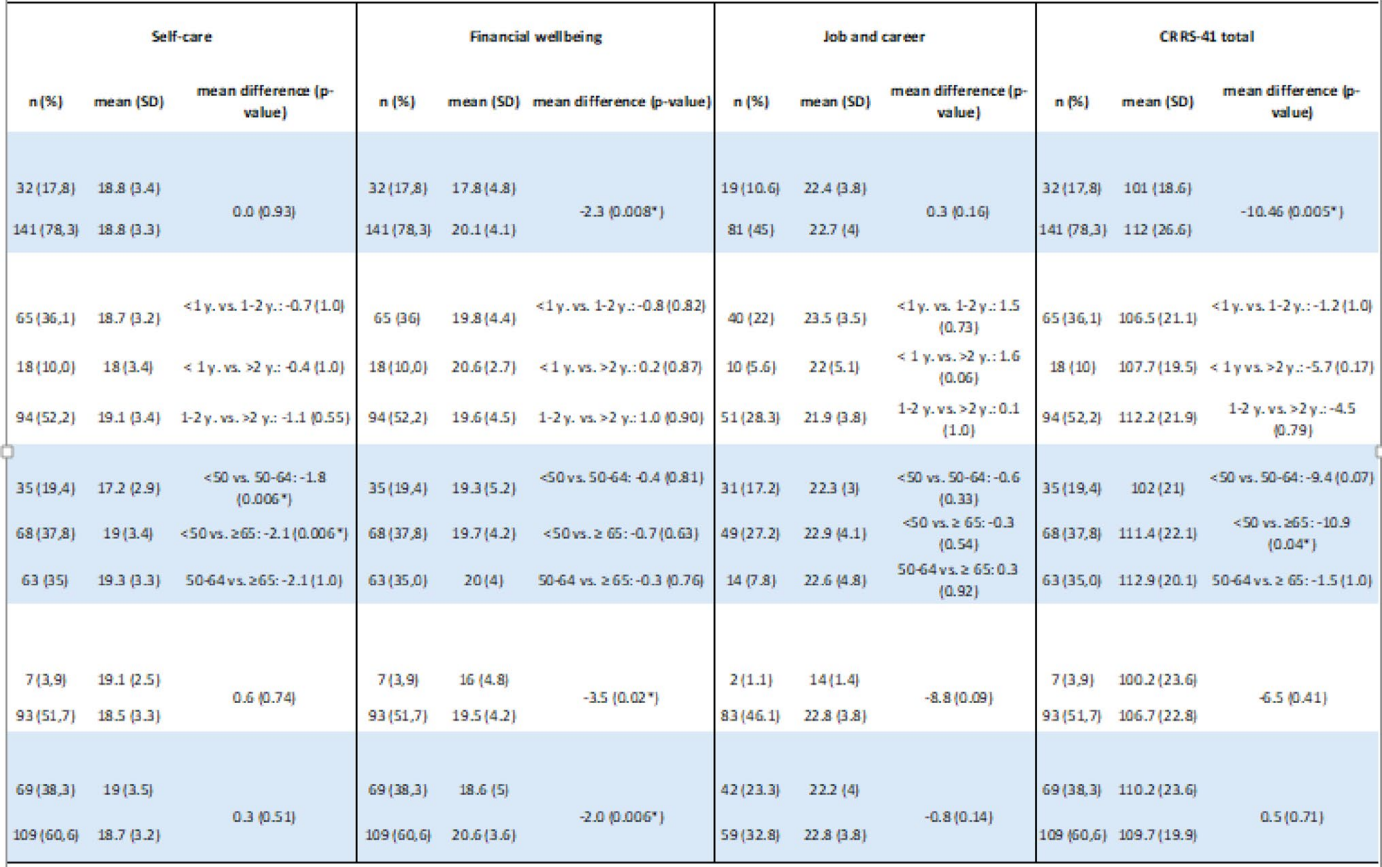
Difference in subscale and CRRS-41 scores between groups by demographic and clinical variables.**

Age was significantly associated with the total CRRS score (*p* = 0.036). Younger caregivers (< 50 years) scored lower in *Emotional Health and Wellbeing* (−3.80, *p* = 0.040) and *Self-care* (−2.07, *p* = 0.006) compared to older caregivers (≥ 65 years) ( −3.80, *p* = 0.040) and (50–64 years) ( −1.87, *p* = 0.006). Caregivers of hospitalized patients scored significantly lower in *Lifestyle* (mean difference: −5.51, *p* = 0.001) and *Emotional Health and Wellbeing* (−3.42, *p* = 0.02) compared to caregivers to a non-hospitalized person. Caregivers whose relatives were diagnosed within the past year had lower scores in *Emotional Health and Wellbeing* compared to those with diagnoses over 2 years ( −3.62, *p* = 0.010). Subscale 5 *Financial Wellbeing* showed significant differences by gender ( −2.04, *p* = 0.006), hospitalization status (−2.30, *p* = 0.020), and employment termination due to caregiving (−3.58, *p* = 0.025).

## Discussion

This study translated and validated the Danish CRRS, demonstrating good internal consistency and test-retest reliability In line with the original CRRS development study the instrument proved useful for assessing the impact of caregiving across cancer types and stages [18].

The translation process underscored the importance of linguistic nuances, to ensure cultural alignment [29]. Participants generally found the items relevant, although some noted that the term ‘responsibilities’ emphasized external caregiving demands rather than emotional commitment. Despite the limited focus on caregiving’s positive aspects, previous studies show that meaningful experiences and perceived competence can buffer negative outcomes of caregiving and bolster resilience [30, 31, 32].

Psychometric evaluation in 180 Danish caregivers showed close correspondence with the original validation. Subscale means and standard deviations were comparable to those previously reported, supporting both sample comparability and consistent scale performance across settings. This similarity suggests that CRRS performs consistently in our caregiver population and captures comparable caregiving response patterns across different settings, with the Danish results largely reproducing the psychometric profile of the original CRRS. The total scale showed excellent internal consistency (α = 0.91), closely matching the original validation (α = 0.92–0.94). Similarly, the subscales *Emotional Health and Wellbeing* (α = 0.87), *Lifestyle* (α = 0.88), *Support and Impact* (α = 0.83), and *Financial Wellbeing* (α = 0.76) showed good internal consistency, in line with earlier findings (0.78–0.87). As in the original study, *Self‑care* (α = 0.68) and *Jobs and Career* (α = 0.54) demonstrated the weakest performance, likely reflecting variability in caregiving and employment contexts. In Denmark supportive welfare and workplace structures may reduce the relevance of work-related caregiving burden, which may affect the applicability of the Subscale 6, *Jobs and Career* for employed caregivers. A larger and more diverse sample could further enhance the reliability of the findings.

In the present study, we evaluated test-retest reliability, and found overall excellent results, suggesting that the measurement produces consistent results across repeated administrations. These findings are consistent with those reported in the original CRRS validation study, thereby further reinforcing its validity. The high ICC value supports the instruments capacity to assess responsiveness to change, confirming its practicality and relevance for evaluating interventions in both clinical and research settings. However, ceiling effects were observed on some items, which may distort the assessment of both test-retest reliability and responsiveness to change. Notably, the item CF4 regarding additional financial costs of supporting the patient showed that more than 90% of participants achieved the maximum score. Skewness in Subscale 5, *Financial Wellbeing*, was also observed in the original validation study, possibly because both Danish and UK patients do not pay for medical treatment [18]. Furthermore, the statistically significant difference between mean scores at t0 and t1 for Subscale 6, *Job and Career*, should be interpreted with caution due to the subscale’s poor internal consistency and test-retest reliability.

In addition, we examined how changes in CRRS scores from t0 and t1 related to caregivers’ self-perceived changes in responsibilities. We found that higher total CRRS scores were significantly associated with self-perceived improvement, indicating that increases in wellbeing measured by the CRRS align with caregivers’ own assessment and supporting the scale’s responsiveness. Exploratory post hoc group comparisons showed clinically plausible differences in subscale scores across caregiver characteristics. However, without a priori hypotheses or an external criterion, these findings should be interpreted cautiously and cannot be considered formal evidence of construct or criterion validity.

In exploratory group comparisons, we found that caregivers of hospitalized patients had lower *Lifestyle* and *Financial Wellbeing* subscale scores than those supporting non-hospitalized patients, while younger and less experienced caregivers reported lower *Emotional Wellbeing* scores. These findings are consistent with previous research highlighting the heightened distress in caregivers during active treatment [3, 33], and greater burden among younger caregivers [34, 35].

### Limitations and future research

While this study provides valuable insights into the psychometric properties of the CRRS in a Danish context, it is important to acknowledge some limitations.

The pilot-test sample consisted mainly of female spousal caregivers, limiting diversity. Although the pilot aimed to assess face validity and item clarity rather than represent all caregiver types, the small purposive sample may not fully reflect the Danish caregiver population. In the psychometric sample most participants were spouses or partners (71%) as patients were asked to nominate their primary caregiver, thereby limiting variation. Future studies should include underrepresented groups such as adult children, who often carry substantial caregiving responsibilities [36].

Because no external criterion was available or no a priori hypotheses for known-groups comparisons were specified, construct and criterion validity could not be formally evaluated according to COSMIN standards. The lack of validated instruments also precluded assessment of convergent validity. These exploratory findings should therefore be interpreted with caution, and future research should address these gaps. However, the observed associations with age and hospitalization status in this study may help inform the development of a priori hypotheses to guide future construct validity testing. Such hypotheses could clarify whether these variables consistently relate to the subscales in theoretically expected ways, thereby strengthening the evidence for construct validity in subsequent studies.

Integrating caregiver self-reported outcomes into clinical care could improve understanding of caregiver needs and support targeted interventions.

## Conclusion

This study successfully translated and validated the Danish version of the CRRS, confirming its satisfactory psychometric properties, based on internal consistency, test-retest reliability, and exploratory associations with relevant caregiver characteristics, for assessing the impact of cancer caregiving on informal caregivers’ quality of life. The psychometric evaluation of the Danish CRRS, based on data from 180 participants, provided valuable insights into caregivers supporting individuals with various cancer diagnoses and demonstrated that the tool is valid and reliable for assessing caregiver burden and wellbeing in both single and longitudinal measurements within research and clinical cancer settings. Limitations include a predominantly spousal caregiver sample, and future research should target a more diverse caregiver population, including non-partner caregivers.

## Data Availability

The data supporting the findings of this study are not publicly available, but they can be accessed upon reasonable request by contacting the corresponding author.
